# Presentation and management of delayed bronchopleural fistula after pulmonary lobectomy: a case report

**DOI:** 10.1186/s13019-024-02795-8

**Published:** 2024-05-31

**Authors:** Riad Abdel Jalil, Ali Dabous, Almu’atasim Khamees, Ahmad Yasin Alzu’bi, Joud Al-Majali

**Affiliations:** 1 Department of General Surgery, King Hussein Cancer Center, Amman, Jordan; 2 Department of Surgery, King Hussein Cancer Centre, Amman, Jordan

**Keywords:** Lung, Resection, Delayed air leak, Cancer, Lobectomy, Robotic, Thoracic, Surgery

## Abstract

**Background:**

Lung cancer is the second most diagnosed cancer and the leading cause of cancer deaths worldwide. Surgical lung resection is the best treatment modality in the early stages of lung cancer as well as in some locally advanced cases. Postoperative air leak is one of the most common complications after pulmonary resection with incidence ranging between 20 and 33%. The majority of air leaks seal, within 5 days after surgery, on their own by conservative management. However, at least 5% of patients still have prolonged air coming out from the residual lung at discharge. This report describes the management of a thin lady with right lung cancer who underwent a right lower lobectomy and then suffered from a delayed air leak 7 weeks after surgery and required extensive thoracic and general surgery collaboration.

**Case presentation:**

A 72-year-old heavy smoker female patient diagnosed with stage I lung cancer underwent right robotic-assisted thoracoscopic surgery converted to thoracotomy because of a fused fissure, right lower lobectomy, and mediastinal lymphadenectomy presented with delayed air leak 49 days after surgery. VATS decortication and mechanical pleurodesis were done 2 weeks after unsuccessful conservative treatment. Still, the lung failed to expand four weeks later so the patient was sent to surgery; she is underweight (BMI of 18) with not many options for a big flap to fill the chest cavity empty space. Accordingly; the decision was to use multiple pedicle flaps; omentum, intercostal muscle, and serratus anterior muscle to cover the bronchopleural fistulas and fill the pleural space in addition to mechanical and chemical pleurodesis. Full expansion of the lung was obtained. The patient was discharged on Post-Operative day 5 without remnant pneumothorax.

**Conclusions:**

Air leaks After lobectomy usually presents directly postoperatively; various management options are available ranging from conservative and minimally invasive to major operative treatment. We presented what we believe was unusual delayed bronchopleural fistula post-lobectomy in a thin lady which demonstrates clearly how a delayed air leak was detected and how collaborative efforts were crucial for delivering high-quality, safe, and patient-centered care till treated and complete recovery.

## Background

Cancer is becoming more common, and with that comes a wide range of treatment options and complications; this proves the importance of a multidisciplinary team. Lung cancer is the second most diagnosed cancer worldwide (after breast cancer), and it is by far the leading cause of cancer deaths accounting for about 1.80 million deaths in 2020 [1]. Each year, more people die of lung cancer than of colon, breast, and prostate cancers combined [2].

Postoperative air leak is one of the most common complications after pulmonary resection and contributes to postoperative pain, complications, and increased hospital length of stay [3]. Several risk factors, including both patient and surgical characteristics, increase the frequency of air leaks [3]. Air leak rates have varied in the literature, but the largest series report a rate between 20 and 33% after elective pulmonary resection, with a majority of air leaks sealing on their own, within 5 days of surgery and are managed with tube thoracostomy and observation [4]. However, prolonged air leak (PAL) is considered if the leak present for more than five to seven days [5].

It has been reported that although specific techniques, intraoperative strategies, and dedicated devices are commonly used to prevent prolonged air leaks (PAL)—at least 5% of patients still present air coming out from the residual lung at discharge [6]. This report describes the management of a lady with right lung cancer that suffered from a delayed air leak and required extensive thoracic and general surgery collaboration. The case demonstrates clearly how a delayed air leak was detected and how collaborative efforts were crucial for delivering high-quality, safe, and patient-centered care.

## Case presentation

A 72-year-old female patient, who has a prolonged history of diabetes mellitus type 2, hypertension, and was a heavy smoker of 50 pack-year, was accidentally found to have a 2 cm nodule in the right lung lower lobe. She was then referred to King Hussein Cancer Center (KHCC) for further investigation. A Positron Emission Tomography Computerized tomography (PET CT) scan was done and showed a peripherally located sub-solid cavitary lesion in the right lower lobe posteriorly measuring about 2.5 × 2 cm in maximum axial dimensions, with no evidence of other concerning hypermetabolic lesions. Computerized tomography (CT)-guided biopsy by interventional radiology was done and pathology examination showed well-differentiated adenocarcinoma with lepidic pattern. The staging was T1N0M0. The multidisciplinary committee (MDC) recommendation was to go with surgical intervention.

Normal preoperative routine labs with a pulmonary function test (PFT) resulted in FEV1 at 68% and DLCO at 95%. On the 19th of October 2022, the patient underwent right robotic-assisted thoracoscopic surgery (RATS), which showed difficulty in dissecting right fused pulmonary fissures. Accordingly, robotic surgery converted to anterolateral thoracotomy with right lower lobectomy and mediastinal lymphadenectomy. The pathology report showed completely resected 2.2 cm well-differentiated adenocarcinoma, and pathology staging was reported as pT2N0; due to visceral pleural invasion. Negative Lympho-Vascular Invasion (LVI).

The postoperative (post-op) course was smooth, she had one chest tube, the pain was controlled, and she used the incentive spirometer efficiently. Daily chest x-ray showed good right lung inflation. The chest tube was removed on postoperative day four and the patient was discharged home assuring compliance for incentive spirometer use. 49 days after the first surgery, she presented to the Thoracic surgery clinic with a 7-day history of shortness of breath, chest CT scan showed a new large right pneumothorax with a small dependent pleural fluid resulting in segmental pulmonary atelectasis. A suspicious focal connection between the right lower bronchus stump and pleural cavity was suspected suggesting a bronchopleural fistula (BPF).

**Fig. 1:**
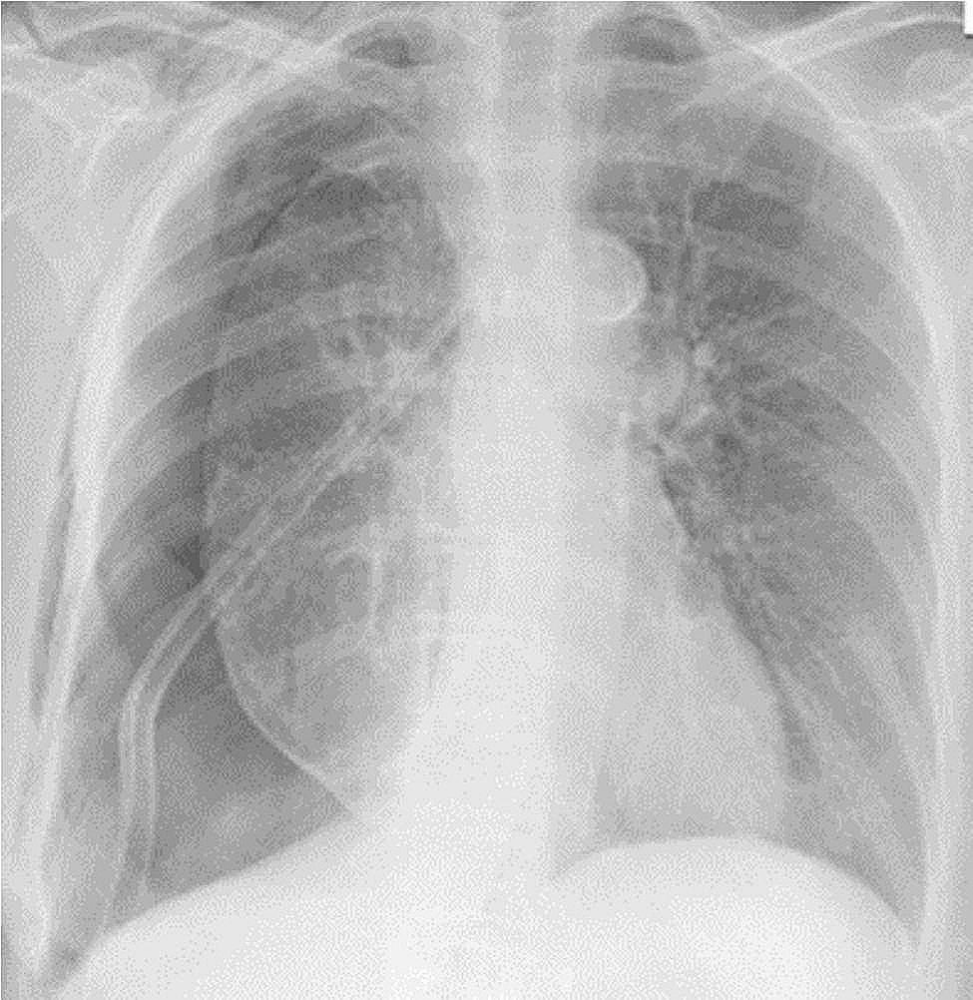
The patient’s chest x-ray before the third surgery

The patient was admitted, and a pigtail chest drain was inserted right away and connected to the chest bottle on wall suction, around 200 ml of serosanguinous fluid was withdrawn at insertion with continuous air bubbling seen. On day 3 post-admission she was doing well, with no shortness of breath reported, vital signs were within normal limits, the pigtail drain was connected to the Heimlich valve, and the patient was discharged home. After one week of follow-up, a chest x-ray showed persistent right pneumothorax without improvement in the right lung inflation. Conservative treatment continued for another week but the chest x-ray didn’t show any changes. Accordingly, the thoracic surgery team decided to go for the right single-port VATS decortication and pleurodesis which was done utilizing a single-port VATS approach through the previous chest tube wound. After that, the patient was observed in the hospital for four weeks with not much improvement in lung expansion (Fig. 1). Bronchoscopy was done by the interventional pulmonary team in the hope to locate the bronchial fistula by occlusion of different sub-segments from the upper and middle lobes but couldn’t localize the fistula exactly, a glue was installed over the lower lobe stump site.

103 days after the first operation, the patient was sent to surgery again. because she is thin (Body Mass Index (BMI) is 18) and there are not many options for a big flap to fill the chest cavity empty space, we decided to use multiple flaps; the general surgery team performed laparoscopic surgery in which the greater omentum was transferred as a pedicle flap passing through an opening in the right hemi diaphragm anterior to the liver to the right chest cavity, this procedure was assessed by redo right VATS, which was converted to mini- anterolateral thoracotomy to extract the omentum. Pleural decortication and closure of multiple bronchopleural fistulas with an omental flap were done. Intercostal muscle and serratus muscle pedicle flaps were used to aid with pleural space-filling and BPF coverage in addition to mechanical and chemical pleurodesis. Full expansion of the lung was obtained.

**Fig. 2:**
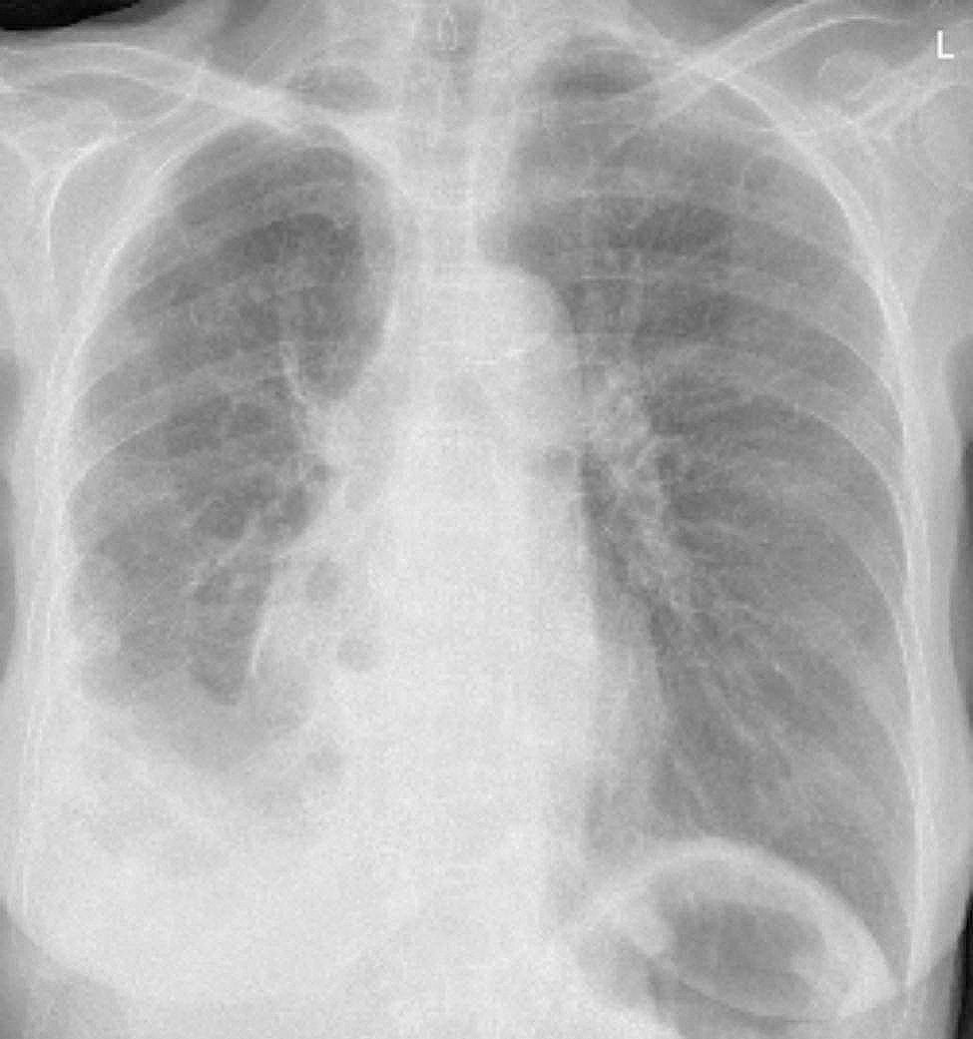
Her chest x-ray after her third operation on postoperative day 1

**Fig. 3:**
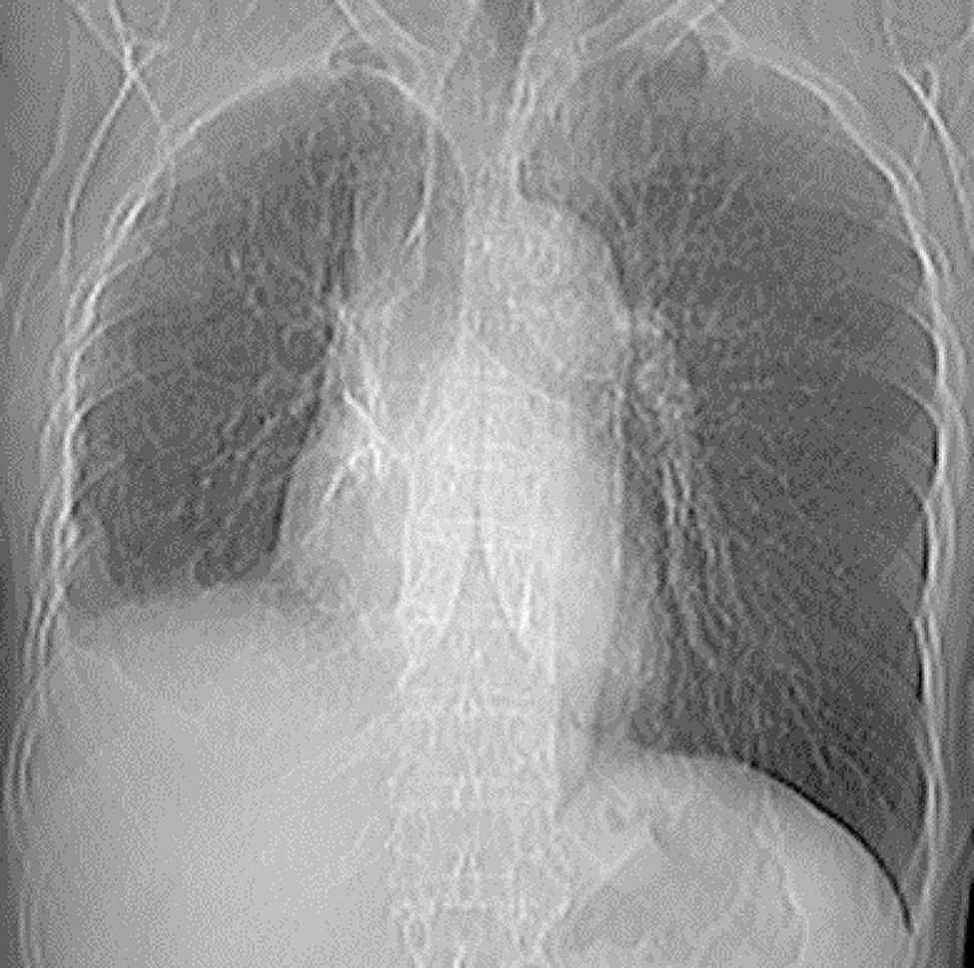
Her follow-up chest x-ray after 6 months of the third operation

The patient spent the night in the intermediate surgical unit and was transferred to the floor on the second-day post-op. She had two chest tubes; one anteriorly and the other posteriorly on the right side. She had a daily chest x-ray with good expansion and no air leak (Fig. 2). The post-op course was smooth, she was doing well without any signs of respiratory distress. On day 4 post-op the anterior chest tube was removed and there was no air leak, the posterior chest tube showed a slight air leak. On day 5 post-op there was no air leak, and the posterior chest tube was removed as well and the patient was discharged home. The patient was followed up in the thoracic surgery clinic, she was doing well, chest x-ray showed excellent right lung expansion without remnant pneumothorax. Also, there were no recurrent air leaks after a six-month (Fig. 3) and one-year follow-up.

## Discussion

The use of robotic systems in healthcare and especially surgical procedures has rapidly increased in the last decade. This lady was the first patient to undergo a robotic-assisted lobectomy in Jordan. According to Yang et al., robotic-assisted thoracic surgery was advantageous in a case of lobectomy in a cancer patient with situs inversus totalis as it helped in identifying the anatomical abnormalities and facilitated the surgery [7]. In addition, Nomori et al. concluded that a largely fused fissure is not a limiting factor for the performance of VATS lobectomy [8]. While in our case it was challenging to complete the surgical procedure with fused pulmonary fissures and difficult dissection therefore the conversion to open lobectomy was mandatory.

Air leaks after elective pulmonary resection occur in approximately 28–60% of patients, with most of them resolving by postoperative day four [9]. Regarding air leaks occurring post-lobectomy, there was no case of delayed air leaks occurring more than a month after surgery in the literature review. The literature discussed prolonged persistent air leaks extensively, but none mentioned cases that were timely remote from surgery. Our patient complained of her first symptom of shortness of breath 49 days postoperatively despite having chest x-rays as an inpatient and outpatient during follow-up.

The management of air leaks varies from conservative by chest drain to chemical pleurodesis and surgical intervention. We attempted chest drain insertion and VATS decortication and pleurodesis without benefits. Thereafter bronchoscopy was done by the pulmonary team with the appliance of endobronchial glue. Despite this, the patient still suffered from a continuous right pleural hydro-pneumothorax. Although Kapicibasi & Baysungur proved a success rate of 80% in patients who underwent bronchopleural fistula repair using N-butyl cyanoacrylate by an endoscopic method [10].

Muscle flaps can be used in thoracic surgery to reconstruct the defect with well-vascularized tissue, space-filling, separate tissues in some cases, and closure of fistulas or stumps to prevent air or fluid leaks to the pleural space [11]. However, these flaps are not commonly used unless there is a unique issue [11].

Finally, the greater omentum was transferred as a pedicle flap passing through an opening in the right hemi diaphragm anterior to the liver to the right chest cavity, this procedure was assessed by redo right VATS, which was converted to mini-anterolateral thoracotomy to extract the omentum. Pleural decortication and closure of multiple bronchopleural fistulas with an omental flap were done. Intercostal muscle and serratus muscle pedicle flaps were used to aid with pleural space-filling and BPF coverage in addition to mechanical and chemical (Talc powder) pleurodesis. Full expansion of the lung was observed.

Woo et al. presented case series for 5 patients who had air leaks postoperatively, resulting from BPF, with failed prolonged conservative management. He used latissimus dorsi and serratus anterior muscles for fistula repair and closure of rib defect respectively [12]. Moreover, these dual flaps depend primarily on the muscle bulk, which was small in our case, who had below normal BMI. Accordingly, we use triple flaps for the closure of the BPF.

## Conclusions

Air leaks post-lobectomy usually present directly postoperatively; various management options are available ranging from conservative and minimally invasive to major operative treatment. We presented what we believe was unusual delayed bronchopleural fistula post-lobectomy which demonstrates clearly how a delayed air leak was detected and how collaborative efforts were crucial for delivering high-quality, safe, and patient-centered care till treated and complete recovery.

## Data Availability

This will be available upon request.

## Abbreviations

KHCC: King Hussein Cancer Center
PET: Positron Emission Tomography
CT: Computerized tomography
MDC: multidisciplinary committee
PFT: pulmonary function test
LVI: Lympho-Vascular Invasion
VATS: Video-assisted thoracoscopic surgery
post-op: postoperative
BPF: bronchopleural fistula
BMI: Body Mass Index
